# Clinical performance of quantitative PCR for the molecular identification of skeletal tuberculosis from formalin-fixed paraffin-embedded tissues

**DOI:** 10.1186/s12879-022-07641-7

**Published:** 2022-07-28

**Authors:** Gang He, Chun-yu Chen, Xin Zhang, Pei-pei Ding, Chang-zheng Hu, Xiu-fang Huang, Xian Zhang, Xu Gong, Pei-lin Zhen, Liang Zhang

**Affiliations:** 1 Department of Infection, Jiangmen Central Hospital, Jiangmen, 529000 China; 2 Department of Infection, Guangdong Medical University, Zhanjiang, 524000 China; 3 Department of Science and Education, Jiangmen Central Hospital, Jiangmen, 529000 Guangdong China; 4 Translational Medicine Center, Guangdong Women and Children Hospital, Guangzhou, 511400 China

**Keywords:** Skeletal tuberculosis, qPCR, Acid fast staining, paraffin specimen

## Abstract

**Background:**

At present, skeletal tuberculosis (TB) diagnosis is mostly by histopathology, but the positivity rate is low. There is a need to develop new methods for the molecular identification of this disorder. Therefore, we aimed to investigate the clinical utility of quantitative PCR (qPCR)-based diagnosis of skeletal TB from formalin-fixed paraffin-embedded (FFPE) tissues and its comparative evaluation with acid-fast bacillus staining (AFS).

**Methods:**

We detected *Mycobacterium tuberculosis* (*M. tuberculosis/*MTB) DNA using qPCR and AFS in FFPE tissue samples from 129 patients suspected of having skeletal TB. The sensitivity, specificity as well as area under the curve (AUC) of qPCR and AFS were calculated. Meanwhile, some factors potentially affecting qPCR and AFS results were investigated.

**Results:**

Overall, qPCR outperformed AFS in detecting *M. tuberculosis*. The AUC of qPCR was higher than that of AFS (0.744 vs.0.561, *p* < 0.001). Furthermore, decalcification of bone tissues did not affect the sensitivity and specificity of qPCR tests. Whereas it impacted the performance of AFS, decalcification increased AFS's specificity and decreased its sensitivity (p < 0.05). Moreover, qPCR had a significantly larger AUC than AFS in decalcified and non-decalcified groups (0.735/0.756 vs. 0.582/0.534, *p* < 0.001) respectively. Similarly, the AUC of PCR was more extensive than that of AFS regardless of skeletal TB patients with concomitant pulmonary TB or not (0.929 vs. 0.762; 0.688 vs. 0.524, *p* < 0.01).

**Conclusions:**

Our data demonstrate that qPCR offers superior accuracy for the detection of mycobacteria in FFPE tissues compared to traditional AFS, indicating its clinical value in osteoarticular TB diagnosis.

**Supplementary Information:**

The online version contains supplementary material available at 10.1186/s12879-022-07641-7.

## Background

Tuberculosis (TB) remains a major global public health problem, bone and joint involvement by *Mycobacterium tuberculosis* account for 2.2–4.7% of all TB cases in Europe and 10–15% of extrapulmonary TB cases in the United States [1]. In China, only a few studies described reports on skeletal TB epidemiology. In addition to causing general tuberculous symptoms, skeletal TB invades the cones to cause disc damage and may result in paraspinal or spinal abscesses or even paralysis [2]. Any bone, joint, or spine can be infected by tuberculous bacilli, but the spine, hip, and knee are predilection sites for infection, accounting for 70–80% of bone and joint TB. Clinical and radiological manifestations of skeletal TB are not adequately specific (shared by other lesions) or sensitive, particularly for the atypical cases [1, 3]. Conventional microbiological methods such as acid-fast stained smears for diagnosing TB require 5000–10,000 bacilli per ml for a positive result [4]. Nevertheless, most extra pulmonary specimens do not have such high concentrations of bacilli, thus resulting in low sensitivity [5]. Culture is considered the gold standard for diagnosing of human TB, but it is time-consuming and generally takes 6–8 weeks, given the slow growth of mycobacteria. In addition, diagnosis of extrapulmonary TB, such as skeletal TB, is still challenging because of fewer mycobacteria in non-respiratory specimens [6]. Although histopathological examination of samples is routinely conducted after surgery, it requires professional expertise. Misdiagnoses, delayed diagnoses, and inappropriate treatment for skeletal TB patients may lead to skeletal and joint damage and even disability [7]. Therefore, traditional approaches are neither sensitive nor specific enough, and it is imperative to establish highly effective laboratory testing technologies for the early and differential diagnosis of skeletal TB in clinical practice.

With advances in leading-edge technologies, nucleic acid amplification techniques have attracted considerable interest and have been widely used for the molecular identification of mycobacteria. For example, *M. tuberculosis-*specific nucleic acid detection outperformed mycobacterial culture and AFS in sputum smear [8]. Furthermore, with the development of qPCR for sensitive mycobacterial detection, it harbors advantages over conventional PCR in terms of speed, quantification, and avoiding potential cross-contamination [9]. However, few studies were available on the diagnosis of skeletal TB from FFPE samples using qPCR. Here, we assessed the clinical application of qPCR targeting *M. tuberculosis* for diagnosing bone TB in paraffin specimens and compared the sensitivity/specificity/AUC of qPCR versus conventional AFS. Based on our results, qPCR has demonstrated superior performance in FFPE tissues of bone TB.

## Materials and methods

### Patients and specimen collection

One hundred twenty-nine patients with suspicion of osteoarticular TB in the Jiangmen Central Hospital orthopedics department from 2016 to 2020 were included in this study. The enrolled patients suffered from severe bone damage and had to undergo inpatient surgery. Of them, 39 cases were excluded based on diagnostic criteria for skeletal TB, including clinical presentation, pathological examination, radiographic images, and the efficacy of antimycobacterial therapy (6–24 months). The remaining 90 subjects were confirmed to have skeletal TB. After surgery, FFPE tissues were prepared according to standard procedures and serial sections for each case were used for histopathological analysis and DNA isolation. In addition, all samples were subjected to pathological examination, qPCR, and AFS. The study was carried out by the Declaration of Helsinki and approved by the Ethics Committee of the Jiangmen Central Hospital. Informed consent was obtained from all participants.

### DNA extraction

Five slices (6 μm) per case were pooled from paraffin blocks of skeletal tissues for DNA extraction. Genomic DNA was isolated manually using the TIANGEN FFPE DNA Kit (#DP330, TIANGEN Biotech; Beijing, China) according to the manufacturer’s protocol. In brief, the sections were vortexed vigorously in 1 mL xylene for 1 min and then centrifuged at 12,000 rpm for 2 min. After removing xylene, 1 mL absolute ethanol was added and vortexed for 20 s, followed by centrifugation at 12,000 rpm for 2 min. Next, the ethanol was pipetted off, and the Eppendorf tube was left at room temperature to evaporate residual ethanol. Then, 1 mL TE (pH = 9.9) was added and rotated at 95 °C (1000 rpm, 10 min), ended by centrifugation at 12,000 rpm for 2 min. After carefully removing the supernatant, the pellet was collected. The resulting DNA was stored at − 20 °C until use.

### Quantitative PCR (qPCR)

Aliquots from the extracted DNA were submitted to mycobacteria qPCR. We used a commercial kit (Guangdong Bright-Innovation BioMed Co., Ltd., Shunde, China) to target three mycobacterial DNA sequences, IS6110, HSP65, and ITS, respectively, for differential detection of *M. tuberculosis*, *M. abscessus* complex (MABC) and *M. avium* complex (MAC). In fact, DNA sequences of various subspecies of MABC including *M. abscessus*, *M. massiliense* and *M. bolletii* or MAC including *M. intracellulare* and *M. avium*, are highly homologous. In addition, the treatment regimen for patients affected by the different subspecies of MABC or MAC is consistent. Therefore, we designed primers and probes to target MABC and MAC, rather than their subspecies. The qPCR assay consisted of a DNA template, forward primer, reverse primer, probe, and PCR mix buffer in a 25 µL. The cycling conditions shared for three targets were as follows: 1 cycle at 95 °C for 5 min, followed by 45 cycles at 95 °C for 15 s, one cycle at 72 °C for 30 s, and collection of Taqman probe fluorescence signal at 60 °C for 30 s according to the manufacturer’s instructions. Analyses of *M. tuberculosis*, MABC and MAC were separately conducted in three tubes for each DNA sample. A blank control, negative control, and a positive sample were tested in parallel in each qPCR run. The housekeeping gene β-actin (ACTB) was used as an internal reference to control for the absence/presence of inhibitors and the quality of the samples after extraction. The cycle threshold (Ct) values were measured at a fixed fluorescence threshold. Samples with Ct > 40 were considered negative. Repeated experiments were conducted for cases with qPCR-negative but AFS-positive. All analyses were performed on a SLAN®96P Real-Time PCR System (Shanghai Hongshi Medical Technology, China).

### Acid-fast bacillus staining (AFS)

Qualified pathologists used a modified Wade-Fite staining technique for AFS. Briefly, tissue sections (6 μm) were oven-baked at 65 °C for 2 h and deparaffinized in a mixture of gasoline and turpentine for two changes, 30 min each. Then, sections were rinsed with running water for 5 min and dropped into a basic Fuchsin solution at room temperature for 10 min, followed by washing and differentiation with 20% sulfuric acid for 3 min. As a result, the sections should be pale pink or colorless under microscopy. Finally, sections were counter stained with 0.1% methylene blue, dehydrated in graded alcohols, dipped in xylene to clear, and mounted in neutral gums. Acid-fast bacilli, including leprosy bacilli and tuberculosis bacilli, were colored bright red, nuclei, and background were stained blue.

### Statistical analysis

Statistical analyses were calculated using the SPSS 26.0 software. The Mann–Whitney U test was used for continuous variables between two groups. All tests were two-sided, and *p* < 0.05 was considered statistically significant. To assess the sensitivity and specificity of qPCR along with AFS and further compare their ability to diagnose skeletal TB, we constructed receiver operating characteristic (ROC) curves and calculated the area under the curve (AUC) using deLong's test of the R Programming Language. The percentage of mycobacteria DNA relative to human cell DNA was calculated using the following formula: 2^(Ct ACTB − Ct Target)^ × 100 [10].

## Results

### Baseline characteristics of the participants

Skeletal TB patients were aged 22–83 years with a median age of 57; 55 cases were males, and 35 were females. Among these patients, the lumbar spine was the most frequently invaded by *M. tuberculosis*, and wrist, elbow, finger, and ankle joints outside the spine were less common. There were 23 bone TB cases with additional pulmonary TB. None of the cases had co-infection with human immunodeficiency virus (HIV). The baseline characteristics of patients are given in Table 1.

**Table 1:** Clinical information of the included subjects

	Number of cases
Total number of cases	129
Cases of skeletal TB	90
Gender	
Male	55
Women	35
Median age	57 (22–83)
Lesions involved	
Lumbar spine	44
Thoracic spine	29
Cervical spine	6
Knee	4
Hip	4
Wrist	1
Elbow	1
Ankle	1
Pulmonary TB	23
HIV positive	0

### QPCR outperformed AFS in detecting *M. tuberculosis*

**Fig. 1:**
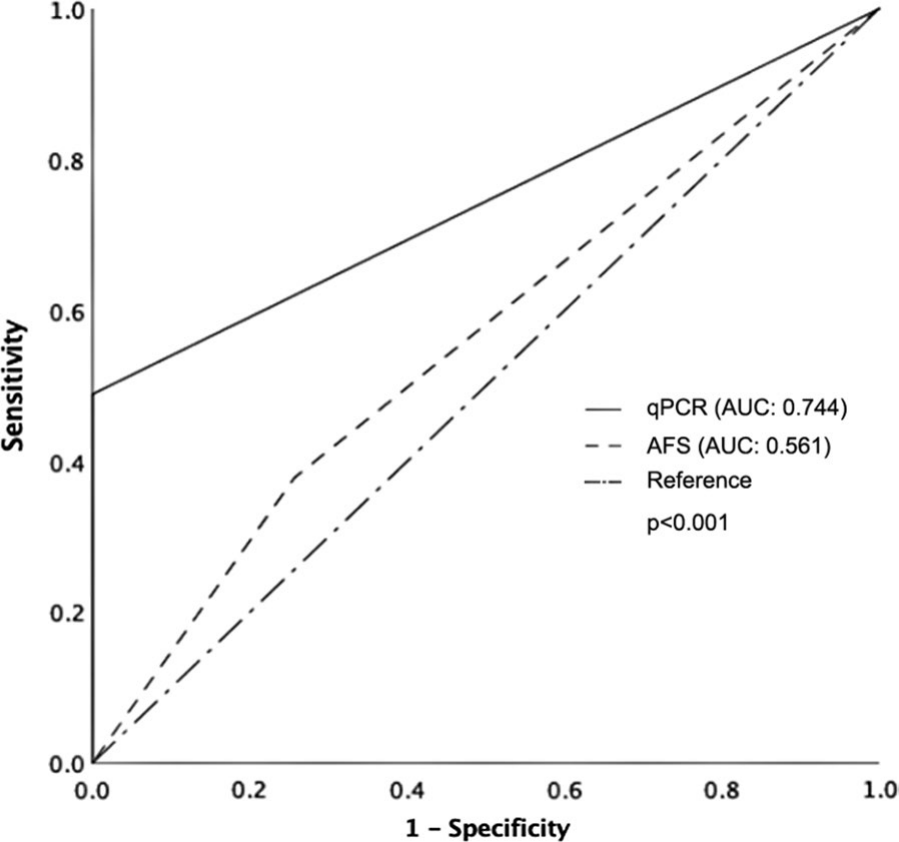
ROC curve analysis showed the performance of qPCR vs. AFS in diagnosing skeletal TB

Of the 129 cases included in this study, 90 subjects were diagnosed with skeletal TB. In the present study, qPCR targeting *M. tuberculosis* has a sensitivity of 48.88% and a specificity of 100%. For AFS, the corresponding data were 37.77% and 87.17%, respectively (Table 2). To evaluate the clinical performance of qPCR for diagnosing skeletal TB, we compared the ROC/AUC between qPCR and AFS (Fig. 1). The AUC analyses of qPCR and AFS in skeletal TB patients were 0.744 and 0.561, respectively (*p* < 0.001).

**Table 2:** Comparison of AFS and qPCR for detecting *M. tuberculosis* in 129 bone samples

Methods	TP	FN	TN	FP	Sensitivity (%)	*p*-value	Specificity (%)	*p*-value	PPV (%)	NPV (%)
AFS	34	56	29	10	37.77		87.17		77.27	34.11
qPCR	44	46	39	0	48.88	0.175	100	0.280	100	45.88

*TP* true positive, *FN* false negative, *TN* true negative, *FP* false positive, *PPV* positive predictive value, *NPV* negative predictive value

### Effect of decalcification of FFPE samples on qPCR and AFS assays

To investigate whether the decalcification of FFPE blocks affects qPCR and AFS tests, paraffin samples were divided into decalcified and non-decalcified groups. The sensitivities and specificities of qPCR and AFS in decalcified and non-decalcified groups are summarized in Table 3. Our data showed that decalcification of bone tissues did not affect the sensitivity and specificity of qPCR tests. However, the observation for AFS was different. Decalcification increases AFS's specificity but decreases its sensitivity (p < 0.05).

**Table 3:** Detection performance of qPCR and AFS in various conditions

	TP	TN	FP	FN	Sensitivity (%)	*p*-value	Specificity (%)	*p*-value
Decalcified group								
qPCR	24	22	0	27	47.05		100	
AFS	13	22	2	38	25.49	0.023	91.67	0.166
Non-decalcified group								
qPCR	20	17	0	19	51.28		100	
AFS	21	17	8	18	53.85	0.821	68	0.01
Spinal TB								
qPCR	38	24	0	41	48.10		100	
AFS	31	24	8	48	39.24	0.262	72.2	0.008
Extraspinal TB								
qPCR	6	15	0	5	54.45		100	
AFS	3	15	2	8	27.24	0.139	88.2	0.170
Skeletal and pulmonary TB								
qPCR	18	2	0	3	85.71		100	
AFS	11	2	0	10	52.3	0.019	100	0.301
Skeletal TB alone								
qPCR	26	37	0	43	52.3		100	
AFS	22	37	10	47	31.88	0.475	78.7	0.003

*TP* true positive, *FN* false negative, *TN* true negative, *FP* false positive, *PPV* positive predictive value, *NPV* negative predictive value. The Chi-squared test was used for the statistical analyses

In addition, ROC curve and AUC analyses were performed. As shown in Additional file 1: Fig. S1, qPCR had significantly larger AUCs than AFS in both decalcified and non-decalcified groups (p < 0.001). In the present study, the decalcification of FFPE samples had a negligible effect on qPCR and AFS assays.

### Comparison of qPCR and AFS assays in patients with spinal TB and extraspinal skeletal TB

Skeletal TB is more likely to occur in the spine, possibly due to its high mycobacteria content, and clinical diagnosis of nonspinal skeletal TB is quite tricky. We, therefore, investigated whether the qPCR testing was affected by the affected sites. The sensitivities of qPCR and AFS in spinal TB patients were 48.10% and 39.24%, and the specificities were 100% and 72.2%, respectively (Table 3). For extraspinal skeletal TB subjects, the sensitivities of qPCR and AFS were 54.45% and 27.24%, and the specificities were 100% and 88.2%, respectively. Regarding AUC, Additional file 1: Fig. S2 demonstrated qPCR outdid AFS in diagnosing bone TB in spinal and extraspinal skeletal TB patients (*p* < 0.05).

### Performance of qPCR and AFS assays in skeletal TB patients and cases with concomitant pulmonary TB

The sensitivities of qPCR and AFS in skeletal TB patients with concomitant pulmonary TB were 85.71% and 52.3%, respectively, and the specificity of both assays was 100%. In contrast, the sensitivities of qPCR and AFS in bone TB patients without pulmonary TB were 52.3% and 31.88%, and the specificities of the two methods were 100% and 78.7%, respectively. Also, the AUC of PCR was more significant than that of AFS regardless of whether skeletal TB patients with concomitant pulmonary TB or not (*p* < 0.01, Additional file 1: Fig. S3).

### Inferred proportion of mycobacterial DNA relative to human cells in various conditions

One of the advantages of using qPCR is human β-actin gene acts as the internal control gene in parallel. We can avoid the false-negative results caused by inhibitors within samples. For each positive sample containing *M. tuberculosis*, taking β-actin as the internal reference and using the 2^(Ct ACTB−Ct Target)^ × 100 formula, we can estimate the relative proportion of mycobacterial DNA normalized to the house-keeping gene. A greater value indicates more mycobacteria in human tissues and vice versa.

Whether the lesion was in the spine or not, concomitant with pulmonary TB or not, the paraffin specimens were decalcified, and AFS positive or negative, these patients were divided into four subgroups for statistical analyses. It can be seen that the mycobacteria in the combined pulmonary TB case group had a significantly higher mean percentage relative to human cells than the non-combined TB group, with statistically significant differences. However, although the values differed among the other comparison groups, they were not statistically significant (Table 4).

**Table 4:** The estimated proportion of mycobacterial DNA relative to human cells in each subgroup

	Inferred proportion of mycobacterial DNA relative to human cells (%)	Average rank	U test
Grouping by additional TB			
Skeletal TB plus pulmonary TB	79.66	30.53	
Skeletal TB alone	16.29	16.94	*p* = 0.001
Grouping by decalcification			
Decalcification	45.59	23.52	
Non-decalcification	26.21	21.27	*p* = 0.562
Grouping by AFS results			
AFS-positive	23.33	21.68	
AFS-negative	20.32	20.31	*p* = 0.944
Grouping by affected sites			
Spinal TB	39.53	23.58	
Extraspinal skeletal TB	25.70	22.33	*p* = 0.828

## Discussion

Since routine methods for the rapid diagnosis of osteoarticular TB are neither very sensitive nor specific, developing a reliable technique has become increasingly urgent and important. Technical advances now allow the detection of *M. tuberculosis* at the molecular level. In this retrospective study, we evaluated the benefits of qPCR for the diagnosis of skeletal TB in FFPE samples. It can detect a very low amount of tubercular bacilli and quickly establish the diagnosis within several hours, which is particularly suitable for osteoarticular TB due to its paucibacillary nature. Precise diagnosis of skeletal TB benefits timely and appropriate antitubercular treatment and can prevent further joint damage or disability.

Currently, clinicians use pathological examination as a diagnostic standard for TB in clinical practice. However, it remains challenging to distinguish tuberculosis from other granulomatous diseases, such as nontuberculous mycobacteria (NTM), sarcoidosis, leprosy and systemic lupus erythematosus. In particular, NTM is similar to tuberculosis in histology, clinical manifestations and imaging features. NTM is often treated as drug-resistant TB [11]. Moreover, it is also positive for AFS [12]. Clinical suspicion requires confirmatory evidence to solve the dilemma. If misdiagnosed, it will lead to a waste of medical resources and delay the management of patients. The most common NTM in China is the *M. avium* complex [13, 14], and the *M. abscessus* is also a common pathogen of NTM in South China [15]. Of 129 samples, one case had a positive qPCR for MABC in wrist tissue, and then the patient was followed up. After taking antimycobacterial drugs for about 8 months, the patient still had pain in the right wrist. Since the qPCR test for MABC was positive, we adjusted the treatment and prescribed azithromycin-based medication to the patient. Three months later, his wrist pain was fundamentally eliminated. This is another advantage of qPCR. It has high specificity and can differentiate MTB from NTM to avoid misdiagnosis and missed diagnosis [16].

The percentage of mycobacteria relative to human cells in bone TB plus pulmonary TB patients is higher than that in subjects with bone TB alone, so it's clear that *M. tuberculosis* content in skeletal TB is higher in patients with additional pulmonary TB. At the same time, the presence of pulmonary TB and skeletal TB indicates that the activity of tuberculosis is apparent, which may also be why the qPCR sensitivity of the group with pulmonary TB is higher than that of the group without pulmonary TB.

Because there are many calcium salts in some skeletal tissues, which hinders the use of conventional methods to make paraffin sections, the calcium salts must be removed after fixation to soften the tissues before conventional sections can be made [17]. Therefore, skeletal tissue sections are usually decalcified in clinical practice, and the sensitivity of AFS in decalcified tissues is significantly affected. However, specificity in the decalcified group was higher than that in the non-decalcified group, so there was little difference in the AUC of AFS between the two groups. Whereas, the sensitivity of qPCR was not affected by tissue decalcification; the sensitivity of qPCR was higher in the decalcified group than AFS. In addition, the specificity of qPCR was also higher in the non-decalcified group than AFS. Therefore, the AUC of qPCR was superior to AFS in decalcified and non-decalcified groups.

This study has some limitations. First, since mycobacterial culture was not performed in all cases, the final diagnostic criteria of skeletal TB in this study depend on a combination of clinical suspicion, radiographic imaging findings, pathological examination, and post-treatment follow-up, which may not render a definitive diagnosis, and result in a decreased sensitivity of qPCR for skeletal TB detection in FFPE tissues. Second, the currently used qPCR for NTM analysis only detects the MABC and MAC, which account for most of the NTM. Finally, some AFS-positive cases may have other mycobacterial infections, but qPCR could not determine them.

## Conclusions

The present study shows that qPCR is superior to AFS in skeletal TB diagnosis from FFPE tissues, which could act as an add-on testing in cases of suspected osteoarticular TB in clinical settings.

## Supplementary Information

**Additional file 1: Figure S1. **Evaluation of qPCR and AFS for skeletal TB diagnosis by ROC curve analysis in decalcified (A) and non-decalcified subjects (B). **Figure S2. **Evaluation of qPCR and AFS for diagnosing bone TB by ROC curve analysis in spinal TB (A) and nonspinal skeletal TB (B). **Figure S3. **Evaluation of qPCR and AFS for skeletal TB diagnosis by ROC curve analysis in skeletal TB patients plus pulmonary TB (A) and patients with skeletal TB alone (B).

## Data Availability

All data generated or analyzed during this study are included in this published article.

## Abbreviations

qPCR: Quantitative polymerase chain reaction
TB: Tuberculosis
AFS: Acid-fast bacillus staining
MTB: *Mycobacterium tuberculosis*
NTM: Non-tuberculosis mycobacteria
FFPE: Formalin-fixed paraffin-embedded
ROC: Receiver operating characteristic
AUC: Area under the curve
ACTB: β-Actin
MABC: *M. **abscessus* Complex
MAC: *M. avium* Complex

## References

[1] Pigrauserrallach C, Rodríguezpardo D (2013). Bone and joint tuberculosis. Eur Spine J.

[2] Hao Z, Zhang Y, Shen X, Luo C, Wang X (2015). Staged treatment of thoracic and lumbar spinal tuberculosis with flow injection abscess. Int J Clin Exp Med.

[3] Vuyst DD, Vanhoenacker F, Gielen J, Bernaerts A, Schepper A (2003). Imaging features of musculoskeletal tuberculosis. Eur Radiol.

[4] Cheng VC, Yam WC, Hung IF, Woo PC, Lau SK, Tang BS, Yuen KY (2004). Clinical evaluation of the polymerase chain reaction for the rapid diagnosis of tuberculosis. J Clin Pathol.

[5] Kramer N, Rosenstein ED (1997). Rheumatologic manifestations of tuberculosis. Bull Rheum Dis.

[6] Van der Spoel van Dijk A, Mcleod A, Botha PL, Shipley JA, Kapnoudhis MA, Beukes CA (2000). The diagnosis of skeletal tuberculosis by polymerase chain reaction. Cent Afr J Med.

[7] Rajasekaran S, Soundararajan DCR, Shetty AP, Kanna RM (2018). Spinal tuberculosis: current concepts. Glob Spine J..

[8] Pandey V, Chawla K, Acharya K, Rao S, Rao S (2009). The role of polymerase chain reaction in the management of osteoarticular tuberculosis. Int Orthop.

[9] Wei Z, Zhang X, Wei C, Yao L, Gao X (2019). Diagnostic accuracy of in-house real-time PCR assay for *Mycobacterium tuberculosis*: a systematic review and meta-analysis. BMC Infect Dis.

[10] Bu Q, Wang S, Ma J, Zhou X, Hu G, Deng H, Sun X, Hong X, Wu H, Zhang L (2018). The clinical significance of FAM19A4 methylation in high-risk HPV-positive cervical samples for the detection of cervical (pre)cancer in Chinese women. BMC Cancer.

[11] Shahraki AH, Heidarieh P, Bostanabad SZ, Khosravi AD, Hashemzadeh M, Khandan S, Biranvand M (2015). Multidrug-resistant tuberculosis may be nontuberculous mycobacteria. Eur J Intern Med.

[12] Koh W, Yu C, Suh GY, Chung MP, Kim H, Kwon OJ, Lee NY, Chung MJ, Lee KS (2006). Pulmonary TB and NTM lung disease: comparison of characteristics in patients with AFB smear-positive sputum. Int J Tuberc Lung Dis.

[13] Zhang Z, Pang Y, Wang Y, Cohen C, Zhao Y, Liu C (2015). Differences in risk factors and drug susceptibility between Mycobacterium avium and Mycobacterium intracellulare lung diseases in China. Int J Antimicrob Agents.

[14] Hu C, Huang L, Cai M, Wang W, Shi X, Chen W (2019). Characterization of non-tuberculous mycobacterial pulmonary disease in Nanjing district of China. BMC Infect Dis.

[15] Tan Y, Su B, Wei S, Cai X, Yu P (2018). Epidemiology of pulmonary disease due to nontuberculous mycobacteria in Southern China, 2013–2016. BMC Pulm Med.

[16] Kobayashi N, Fraser TG, Bauer TW, Joyce MJ, Hall GS, Tuohy MJ, Procop GW (2006). The use of real-time polymerase chain reaction for rapid diagnosis of skeletal tuberculosis. Arch Pathol Lab Med.

[17] Witter K, Matulová P, Míšek I (2000). The effects of two different decalcified procedures on size and structure of embryonic epithelial tissue in objects prepared for light microscopy. Anat Histol Embryol.

