# Supplementary material for: Clinical performance of quantitative PCR for the molecular identification of skeletal tuberculosis from formalin-fixed paraffin-embedded tissues
Source: BMC Infect Dis. 2022 Jul 28;22:651. doi: 10.1186/s12879-022-07641-7 (PMC9331151; doi:10.1186/s12879-022-07641-7)

Supplementary Material

Figure S1. Evaluation of qPCR and AFS for skeletal TB diagnosis by ROC curve analysis in decalcified (A) and non-decalcified subjects (B).

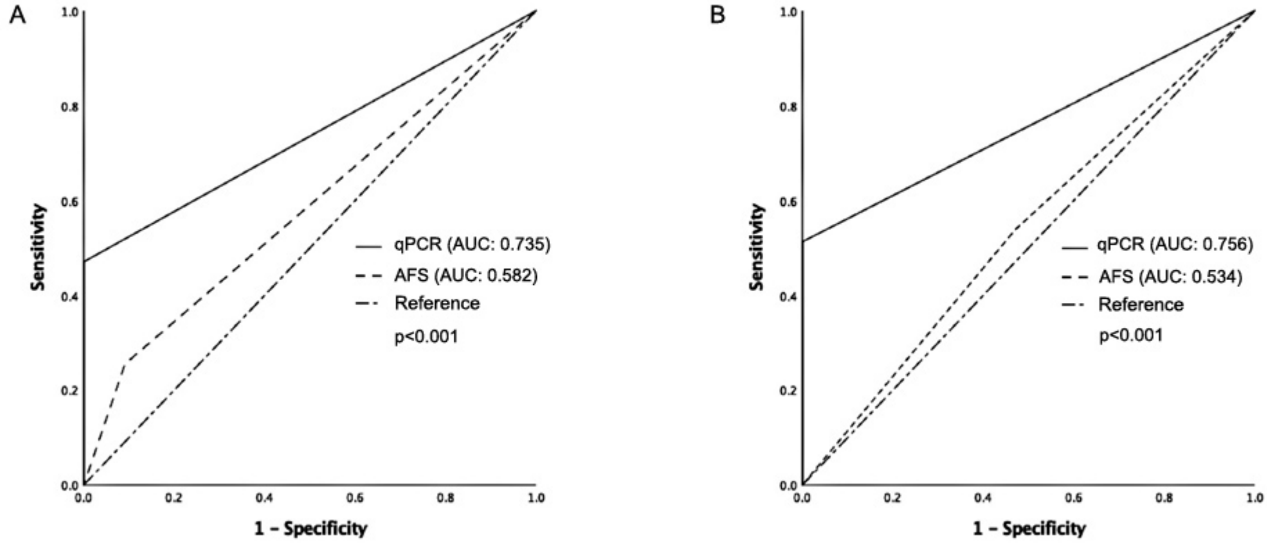

Figure S2. Evaluation of qPCR and AFS for diagnosing bone TB by ROC curve analysis in spinal TB (A) and nonspinal skeletal TB (B).

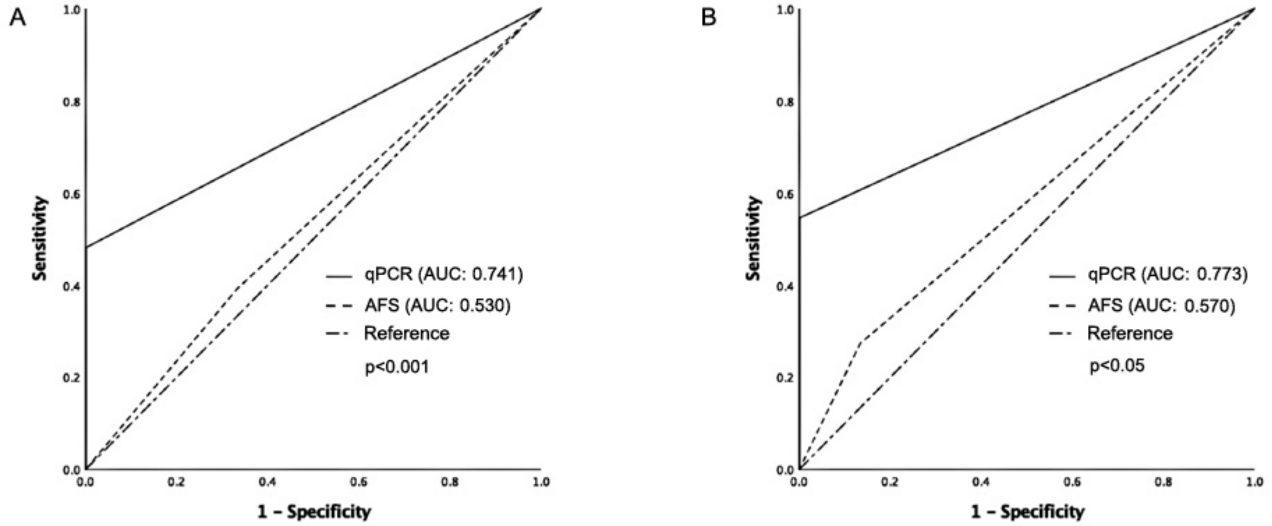

Figure S3. Evaluation of qPCR and AFS for skeletal TB diagnosis by ROC curve analysis in skeletal TB patients plus pulmonary TB (A) and patients with skeletal TB alone (B).

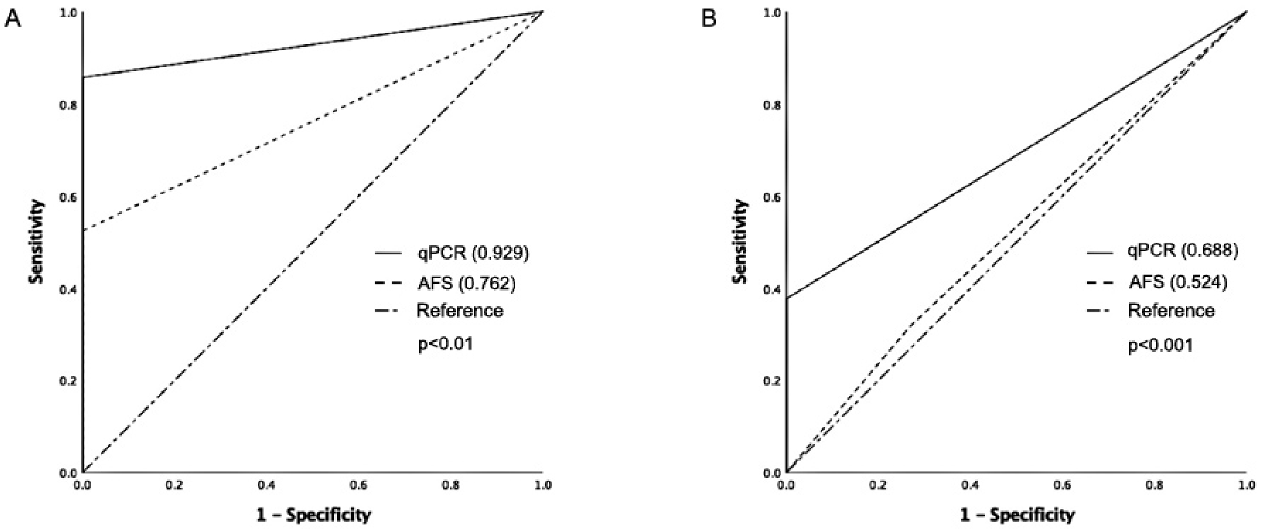

Supplement: Supplementary file 1 — Additional file 1: Figure S1. Evaluation of qPCR and AFS for skeletal TB diagnosis by ROC curve analysis in decalcified (A) and non-decalcified subjects (B). Figure S2. Evaluation of qPCR and AFS for diagnosing bone TB by ROC curve analysis in spinal TB (A) and nonspinal skeletal TB (B). Figure S3. Evaluation of qPCR and AFS for skeletal TB diagnosis by ROC curve analysis in skeletal TB patients plus pulmonary TB (A) and patients with skeletal TB alone (B). [file 12879_2022_7641_MOESM1_ESM.pdf]
